# The incidence and outcome of acute kidney injury during pediatric kidney tumor treatment—a national cohort study

**DOI:** 10.1007/s00467-025-06684-7

**Published:** 2025-02-19

**Authors:** Paulien A. M. A. Raymakers-Janssen, Gerrit van den Berg, Marc R. Lilien, Inge A. van Kessel, Alida F. W. van der Steeg, Marc H. W. A. Wijnen, Mieke I. Triest, Sophie E. van Peer, Marjolijn C. J. Jongmans, Harm van Tinteren, Geert O. Janssens, Marta Fiocco, Roelie M. Wösten-van Asperen, Marry M. van den Heuvel-Eibrink

**Affiliations:** 1 Department of Pediatric Intensive Care, Wilhelmina Children’s Hospital/University Medical Center Utrecht, Utrecht, The Netherlands; 2 Princess Máxima Center for Pediatric Oncology and Wilhelmina Children’s Hospital, Heidelberglaan 25, 3584 CS Utrecht, The Netherlands; 3 Department of Pediatric Nephrology, Wilhelmina Children’s Hospital/University Medical Center Utrecht, Utrecht, The Netherlands; 4 Department of Radiation Oncology, University Medical Center Utrecht, Utrecht, The Netherlands; 5 Mathematical Institute, Leiden University, Leiden, The Netherlands; 6 Department of Biomedical Science, Medical Statistical Section, Leiden University Medical Centre, Leiden, The Netherlands; 7 Wilhelmina Children’s Hospital/University Medical Center Utrecht, Theme Child Health, Utrecht, The Netherlands

**Keywords:** Kidney tumors, Wilms tumor, Acute kidney injury, Chronic kidney disease

## Abstract

**Background:**

Acute kidney injury (AKI) is a serious complication of pediatric cancer treatment that is suggested to increase the risk of chronic kidney disease (CKD). Children with a kidney tumor may be at particular risk. This study aimed to determine the incidence and risk factors of AKI and its association with CKD during pediatric kidney tumor treatment.

**Methods:**

We analyzed data from a prospective national cohort of patients ≤ 18 years old diagnosed with a kidney tumor between 2015 and 2021 in the Princess Máxima Center for Pediatric Oncology in the Netherlands. AKI was defined according to KDIGO criteria. CKD was assessed 1 year post-treatment based on proteinuria and/or decreased estimated glomerular filtration rate (eGFR).

**Results:**

Of 147 patients, we observed AKI in 104 patients (71%) during therapy. AKI occurred most often within 48 h after tumor nephrectomy (88/104), while the rest had non-nephrectomy-related AKI from multifactorial causes. Sixteen patients experienced more than one AKI episode, and 92/104 episodes were reversible. Patients who developed AKI had a higher eGFR prior to surgery compared to those who did not develop AKI. CKD was observed in 16/120 patients (13%). Risk factors for developing CKD included the occurrence of at least 1 AKI event, the use of a > 3-drug regimen, and a lower eGFR at the start of treatment.

**Conclusion:**

The high incidence of AKI and its association with early CKD highlights the need for early detection, prevention, and intervention strategies during pediatric kidney tumor treatment.

**Graphical abstract:**

**Figure:**
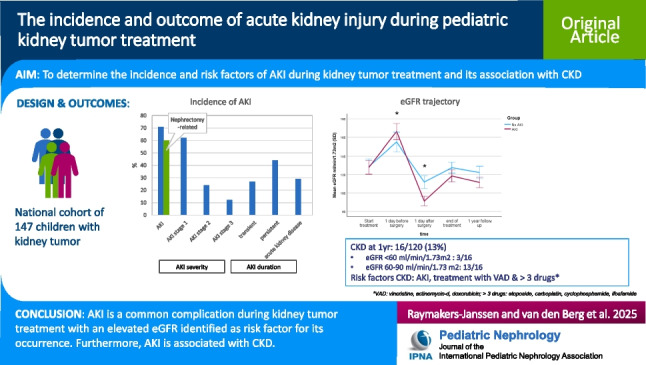
A higher resolution version of the Graphical abstract is available as Supplementary information

**Supplementary Information:**

The online version contains supplementary material available at 10.1007/s00467-025-06684-7.

## Introduction

Kidney tumors are diagnosed in approximately 1200 children annually in Europe [1]. The majority of these tumors are nephroblastomas, also known as Wilms tumors, which generally have excellent survival rates with current response-based treatment strategies. These include, in the International Society of Pediatric Oncology-Renal Tumor Study Group (SIOP-RTSG) setting, multimodality treatment, i.e., pre-operative chemotherapy, followed by tumor nephrectomy, and risk-based postoperative chemotherapy, with or without radiotherapy [2–4]. Given the excellent survival rates, more emphasis is allowed on toxicity. One serious yet somewhat underrecognized side effect that can occur currently during pediatric kidney cancer treatment is acute kidney injury (AKI) [5, 6]. AKI is characterized by a rapid decline in estimated glomerular filtration rate (eGFR) [7]. The etiology of AKI during treatment is multifactorial with nephrotoxic medication (chemotherapeutic agents, antibiotics), decrease of nephron mass by surgery, whole abdomen irradiation, critical circulatory events, infections, the malignancy itself [5–8], and possibly the relatively high prevalence of underlying genetic susceptibility [9], which all may contribute to the development of AKI in kidney tumor patients.

AKI is a clinically significant complication that might require hospitalization and dialysis therapy and can increase morbidity and mortality as a part of multi-organ failure syndrome [10–12]. In certain cases in pediatric oncology, AKI requires postponement or adaptation of chemotherapy, which may impact survival. In addition, patients who experience AKI exhibit an elevated risk of developing chronic kidney disease (CKD) later in life [13, 14], including during childhood [15–17].

The primary reason for CKD in Wilms tumor survivors has been suggested to be nephrectomy [18–21], but proper cohort studies are lacking. After kidney tumor therapy, a significant proportion (14–21%) of survivors suffer from CKD even at a young age [21, 22]. After 35 years of follow-up, the prevalence of CKD among these patients is approximately ten times higher than that observed in their siblings [18]. In pediatric kidney tumor patients, so far, no attention has been paid to the influence of the event of AKI during treatment on the risk of early onset CKD.

Therefore, the primary aim of this study was to determine both the incidence and severity of AKI in a national unselected cohort of patients ≤ 18 years old with a kidney tumor. The secondary aims were to identify risk factors for AKI and to investigate the relationship between the occurrence of AKI during treatment and patients’ variables of CKD 1 year after discontinuation of treatment.

## Materials and methods

### Study population

**Fig. 1:**
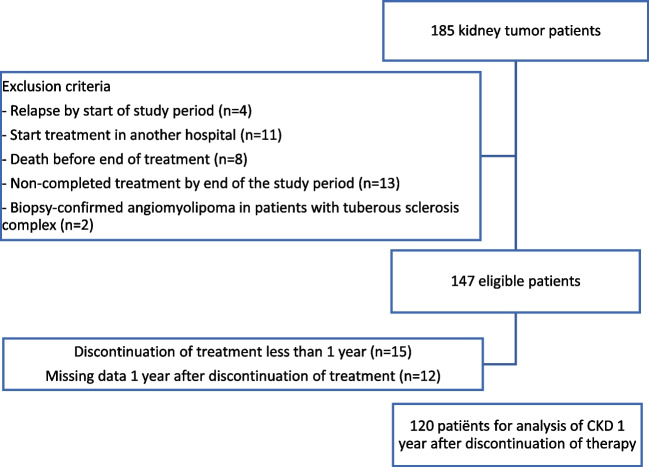
Flow chart of included patients

This cohort study included all Dutch pediatric kidney tumor patients (age ≤ 18 years), who were treated from start to end of protocol between 1 January 2015 and 1 January 2021 in our national pediatric oncology center, the Princess Máxima Center for Pediatric Oncology (PMC) in Utrecht [23]. Exclusion criteria are summarized in Fig. 1. Relevant data were extracted from the electronic medical health records, including demographics (age, sex), kidney tumor characteristics (stage, histology), treatment details (including chemotherapy, supportive care co-medication, radiotherapy, and surgery), and genetic data (germline variant and/or confirmed syndrome).

All patients were treated according to the consecutive SIOP-RTSG clinical trial protocols (SIOP2001) [24] and from 2016 SIOP UMBRELLA [4]. Tumor, demographic, and treatment data were handled in conformity with the General Data Protection Regulation Act, and procedures were approved by the biobanking and data access procedure (BDAC) of the Princess Máxima Center (PMCLAB2019.065). The study was approved by the institutional ethical review board (METC number 19–449/C). Patients and/or parents gave written informed consent.

### Methods

The primary end point of this study was the incidence and severity of AKI. AKI was defined according to the “Kidney Disease: Improving Global Outcomes” (KDIGO) criteria [25], using serum creatinine (sCr) level. sCr was measured daily around nephrectomy and chemotherapy courses. According to this classification, patients with sCr 1.5–1.9 times baseline were considered to have “AKI grade 1,” patients with sCr 2.0–2.9 times baseline were considered to have “AKI grade 2,” and patients with sCr 3.0 times baseline or initiation of kidney replacement therapy or eGFR < 35 ml/min per 1.73 m^2^ or sCr ≥ 353.6 mcmol/l were considered to have “AKI grade 3.” For patients in whom one or more pre-morbid sCr values were available but showed significant fluctuation, the choice of the serum creatinine measurement that best reflected the most appropriate baseline value was adjudicated by an expert clinician [26].

The duration of each AKI episode was measured. We categorized the duration of AKI episodes as transient AKI (< 48 h), persistent AKI (> 48 h), and AKD (acute kidney disease, > 7 days) [26]. A minimum of 48 h is necessary to separate two distinct AKI episodes [26]. Following this categorization, an AKI > 48 h after nephrectomy was indicated as non-nephrectomy-related AKI. The eGFR was calculated using the modified Schwartz formula [27]. The secondary outcome of this study was the presence of CKD 1 year after discontinuation of treatment, which was defined as having an eGFR of less than 90 ml/min/1.73 m^2^ and/or proteinuria (protein-to-creatinine ratio > 20 mg/mmol at age ≥ 2 years and > 50 mg/mmol at age < 2 years). The KDIGO criteria were applied to children from 2 years of age, but there was only one child with CKD at age < 2 years, who was on KRT. Hypertension was defined as at least three consecutive oscillometric measurements of systolic and/or diastolic blood pressure > 95th percentile for sex, age, and height [28] and/or the use of medication for hypertension.

### Statistical analyses

Demographic and tumor and treatment characteristics were listed and summarized descriptively, according to AKI status and CKD status. Categorical data were summarized as frequencies and percentages. For continuous data, median and interquartile range were reported. The chi-square test was used to compare categorical variables and a two-tailed *t*-test or Mann–Whitney test (in case of violation of the normality assumption) for numeric data.

Linear mixed models, which account for the presence of repeated measures, were employed to investigate changes in eGFR at different time points during treatment in patients with or without AKI. Univariate logistic regression models were estimated to investigate the effect of AKI on CKD. Analyses were performed in IBM SPSS Statistics for Windows, version 29 (IBM Corp, Armonk, NY, USA).

## Results

A total of 185 patients were diagnosed with a kidney tumor during the study period. Of those, 147 (68 male, 79 female) were eligible for analysis. The median age of these patients at diagnosis was 35 months (IQR 18–55). Wilms tumor (*n* = 126) was the most common kidney tumor (Table 1, and in more detail Supplemental Table 1). In our cohort, 14 (10%) patients presented with stage V Wilms tumor and 28 (19%) presented with metastatic disease, including three with local stage V. Preoperative chemotherapy was administered in 126 (86%) patients, and 21 (14%) underwent upfront surgery. One hundred thirty-six (93%) patients underwent total nephrectomy, one patient with localized kidney tumor underwent unilateral nephron-sparing surgery (NSS) only, and ten WT patients got bilateral surgery (unilateral nephrectomy and contralateral NSS or bilateral NSS). Forty-eight (33%) patients received abdominal radiotherapy, of which eight (6%) whole abdominal radiotherapy. Ninety-seven (66%) patients were treated with vincristine, actinomycin-D (VA), and 16 (11%) patients with a > 3-drug regimen. Forty (27%) patients had a genetic predisposing factor for kidney tumors of which 10 with a *WT1*-related syndrome and 17 with a Beckwith-Wiedemann spectrum (BWSp). For 120 patients, follow-up data at 1 year after completion of treatment was available.

**Table 1:** Demographic and clinical data of included kidney tumor patients

Variable	All *N* = 147	AKI *N* = 104	Non-AKI *N* = 43
Female sex (%)	79 (54)	59 (57)	20 (47)
Age (months), median (IQR)	35 (18–55)	35 (20–54)	35 (12–68)
Diagnosis			
– Wilms tumor st I–III (%)	84 (57)	62 (60)	22 (51)
– Wilms tumor st IV (including IV + V) (%)	28 (19)	19 (18)	9 (21)
– Wilms tumor st V (%)	14 (10)	12 (12)	2 (5)
– CMN (%)	5 (3)	3 (3)	2 (5)
– CN (%)	5 (3)	0 (0)	5 (12)
– MRTK (%)	2 (1)	1 (1)	1 (2)
– RCC (%)	6 (4)	5 (5)	1 (2)
– Nephrogenic rest (%)	2 (1)	2 (2)	0 (0)
– Metanephric adenoma (%)	1 (1)	1 (1)	0 (0)
Surgery			
– Total nephrectomy (%)	136 (93)	96 (92)	40 (93)
– Bilateral (3 bilateral NSS, 7 NSS + TN) (%)	10 (7)	8 (8)	2 (5)
– NSS only	1 (1)	0 (0)	1 (2)
Radiotherapy (%)	48 (33)	34 (33)	14 33)
– Flank (%)	39 (27)	27 (26)	12 (28)
– Whole abdomen (%)	9 (6)	7 (7)	2 (5)
Treatment			
– VA (%)	97 (66)	69 (66)	28 (65)
– VAD (%)	13 (9)	11 (11)	2 (5)
– > 3 drugs (%)	16 (11)	13 (13)	3 (7)
– Other (%)	21 (14)	11 (11)	10 (23)
Hypertension at diagnosis			
– Yes (%)	70 (48)	46 (44)	24 (56)
– No (%)	55 (37)	40 (38)	15 (35)
– Missing^#^ (%)	22 (15)	18 (17)	4 (9)
Hypertension at the end of therapy			
– Yes (%)	19 (13)	11 (11)	8 (19)
– No (%)	97 (66)	69 (66)	28 (65)
– Missing^#^ (%)	31 (21)	24 (23)	7 (16)
Genetic predisposing factor for kidney tumors^##^ (%)	40 (27)	26 (25)	14 (33)
– *WT1*-related syndrome (%)	10 (7)	6 (6)	4 (9)
– Beckwith Wiedemann spectrum (%)	17 (12)	15 (14)	2 (5)
– Others (%)	13 (9)	5 (5)	8 (19)

*AKI*, acute kidney injury; *CKD*, chronic kidney disease; *IQR*, interquartile range; *VA*, vincristine, actinomycin-D; *VAD*, vincristine, actinomycin-D, doxorubicin; > *3 drugs*, etoposide, carboplatin, cyclophosphamide, ifosfamide; *eGFR*, estimated glomerular filtration rate; *CN*, cystic nephroma; *CMN*, congenital mesoblastic nephroma; *RCC*, renal cell carcinoma; *MRTK*, malignant rhabdoid tumors of the kidney; *TN*, total nephrectomy; *NSS*, nephron-sparing surgery

^#^No fulfillment of definition with 3 blood pressure readings

^##^Every patient (if parents consented) was tested for Beckwith Wiedemann Spectrum and underwent WES-based panel (for Wilms tumor)

### Incidence of acute kidney injury

Seventy-one percent of the patients (104/147) experienced AKI during therapy. Analysis of records of the eight patients who had died during treatment and were excluded from the analysis showed that seven had experienced at least one episode of AKI before death. Demographics and treatment characteristics for patients with or without AKI are presented in Table 1. AKI occurred in 88 of the 104 patients (84%) within 48 h after nephrectomy. Sixteen of 104 patients (15%) had a non-nephrectomy-related AKI, of which five were in the preoperative phase, the others more than 48 h after surgery. The non-nephrectomy-related AKI had diverse causes, including administration of medication (ICE treatment, amphotericin B) and volume depletion due to vomiting, insufficient intake, or gastroenteritis (Supplementary Table 2). The different AKI episodes during treatment and CKD of the Wilms tumors are illustrated in Supplementary Fig. 1.

During the first AKI episode, 62% had AKI grade 1, 24% grade 2, and 12% grade 3 (including 1 patient on kidney replacement therapy). In 41% of the AKI episodes, the duration was 2 to 7 days. Details of AKI severity and AKI duration are summarized in Supplemental Fig. 2. Sixteen patients experienced more than one AKI episode.

**Fig. 2:**
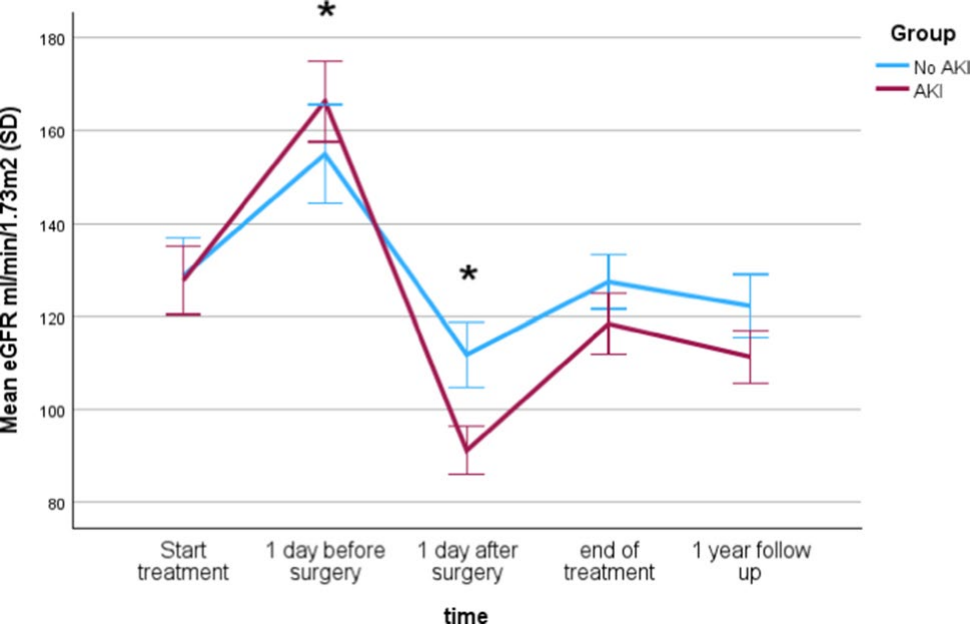
eGFR at different time points during treatment

The trajectory of eGFR during treatment was different between the AKI and non-AKI groups. Patients who developed AKI had higher preoperative eGFR levels as compared to patients without AKI (eGFR 168 ml/min/1.73 m^2^ (SD 34) vs. 150 ml/min/1.73 m^2^ (SD 23) (*p* = 0.001)) (Fig. 2). Linear mixed model shows a significant main effect of time on eGFR (*F* (4353.42) = 131.99, *p* < 0.001). See also the results in Table 2.

**Table 2:** Linear mixed model: changes in eGFR at different time points during treatment in patients with or without AKI

Time	*F*	*p*
Start treatment	0.006	0.936
1 day before surgery	3.909	0.049
1 day after surgery	12.851	< 0.001
End of treatment	2.540	0.112
1-year follow-up	2.061	0.152

*F*-statistics and *p*-values from the linear mixed model examining the effect of time on eGFR. Significant differences (*p* < 0.05) indicate changes in eGFR associated with specific time points

Hypertension at diagnosis was observed in 70/147 (47%) patients. Of these patients, 22 were treated with an ACE inhibitor (enalapril). As ACE inhibitors (ACEi) may decrease eGFR, we performed a sub-analysis for patients using these. There were no differences in eGFR trajectory between patients treated with an ACEi (*n* = 22) and patients not treated with ACEi (*n* = 125). There were also no differences in eGFR trajectory between patients with or without hypertension at diagnosis (data not shown).

### Chronic kidney disease (CKD)

At 1-year follow-up, 16/120 patients (13%) developed CKD (Table 3). Patients with CKD were older (4.6 years), compared to patients without CKD (2.9 years; *p* = 0.04). Of these patients, 15/16 patients had experienced at least one AKI episode during treatment (10 patients had 1 episode of AKI, 3 patients had 2 episodes of AKI, and 2 patients had 3 episodes of AKI). Patients who experienced ≥1 AKI had more frequent CKD (*p* = 0.01). In 13 of these 15 patients, sCr did not return to baseline and AKI was non-reversible. Ten out of 16 (63%) patients with CKD had an AKI episode < 48 h postoperative.

**Table 3:** Characteristics of patients with CKD

Variable	CKD *N* = 16	No CKD *N* = 104	OR (95% CI)	*p*
Age at start diagnosis, years median (IQR)	4.6 (1.7–7.1)	2.9 (1.5–4.7)	1.06 (1.01–1.02)	0.05
eGFR (ml/min/1.73 m^2^)				
– Diagnosis mean (SD)	108 (36)	132 (32)*	0.98 (0.96–0.99)	0.004
– Preoperative (SD)	142 (51)	165 (36)*	0.98 (0.97–0.99)	0.007
– Stop treatment mean (SD)^#^	84 (259)	125 (26)*	0.91 (0.87–0.95)	< 0.0001
– 1 year FU mean (SD)	76 (15)	120 (213)*	0.90 (0.84–0.92)	< 0.0001
Hypertension at diagnosis				
– Yes (%)	3 (19)	53 (51)	0.3 (0.73–1.24)	0.10
– No (%)	7 (43)	37 36)		
– Missing^#^ (%)	6 (38)	14 (13)		
Hypertension end of treatment				
– Yes (%)	2 (13)	14 (13)	2.33 (0.39–14.0)	0.355
– No (%)	10 (63)	75 72)		
– Missing^##^ (%)	4 (25)	15 (14)		
1 AKI during treatment, *n* (%)	15 (94)	70 (67)*	7.29 (1.08–57.4)	0.04
– Duration > 48 h	13	48		
– AKI < 48 h postoperative	10	61		
> 1 AKI episode, *n* (%)	5 (31)	7 (7)		
Radiotherapy (%)	5 (33)	33 (31)	0.98 (0.31–3.04)	0.97
Treatment				
– VA (%)	3 (19)	63 (61)*	0.28 (0.09–0.83)	0.02
– VAD (%)	6 (38)	18 (17)*	3.58 (1.20–10.7)	0.02
– > 3 drugs (%)	4 (25)	10 (10)*	4.53 (1.52–13.5)	0.007
– Other (%)	3 (19)	13 (13)		
Genetic predisposing factor for kidney tumors (%)	4 (25)	27 (26)	1.38 (0.88–2.16)	0.160
– *WT1-*related syndromes (%)	0	7 (7)	N.A	
– Beckwith Wiedemann spectrum (%)	3 (19)	11 (11)	1.95 (0.48–7.93)	0.35
– Others (%)	1 (6)	9 (9)	N.A	
eGFR < 60 ml/min/1.73 m^2^	3			
eGFR 60–90 ml/min/1.73 m^2^	13			

*IQR*, interquartile range; *eGFR*, estimated glomerular filtration rate; *AKI*, acute kidney injury; *VA*, vincristine, actinomycin-D; *VAD*, vincristine, actinomycin-D, doxorubicin; > *3 drugs*, etoposide, carboplatin, cyclophosphamide, ifosfamide; *OR*, odds ratio; *CI 95%*, confidence interval 95%

Univariate logistic regression was used to calculate ORs

*p < 0.05

^#^1 dialysis patient is excluded

^##^No fulfillment of definition with 3 blood pressure readings

In the 16 patients with CKD, eGFR was lower both at the start of treatment (mean 108 ml/min/1.73 m^2^; SD 36) and at the end of treatment (mean 84 ml/min/1.73 m^2^; SD 25) compared to patients without CKD (start; 132 ml/min/1.73 m^2^; SD 32, *p* = 0.006; end 125 ml/min/1.73 m^2^; SD 26, *p* =  < 0.001). Three patients had an eGFR < 60 ml/min/1.73 m^2^ 1 year after completion of therapy. One of these patients already presented with CKD (Stage 1) at diagnosis due to congenital abnormalities of the kidney and urinary tract (FANC/BRCA2-mutation) and experienced two episodes of AKI. Another one had WT stage 2 without other phenotypic features or known germline mutation upon testing by WES and started with a normal eGFR. This patient experienced hypoperfusion of the contralateral kidney due to vascular abnormalities and had severe AKI requiring dialysis for 3 days. The third patient had a mixed-type CMN in a solitary functioning kidney. After NSS, she developed AKI stage 3 for which she was treated by acute hemodialysis for 19 days, followed by peritoneal dialysis until she had a kidney transplant. There was no association between the development of CKD and hypertension, radiotherapy, and/or a genetic predisposing factor for kidney tumors.

Patients with CKD had been treated more frequently with VAD, high-risk protocol, or > 3 drugs compared to patients who were treated with VA only (*p*-value 0.02; *p*-value 0.007).

## Discussion

This study describes a comprehensive evaluation of the incidence of AKI in a substantial longitudinal cohort of 147 patients aged ≤ 18 years with a kidney tumor. Our findings indicate that 71% of the patients experienced at least one AKI episode during treatment, with the highest incidence occurring within 48 h following nephrectomy. A higher baseline eGFR was identified as a risk factor for the development of AKI. Moreover, we showed that the occurrence of at least one AKI episode is associated with an increased risk of CKD, which was observed in 13% of patients within 1 year after completing therapy.

This is the first study that examined AKI throughout the course of pediatric kidney tumor treatment. The high incidence of AKI was mainly observed shortly after tumor nephrectomy (60%, 88/147). This finding aligns with data from a smaller single-center cohort in which an incidence of AKI after nephrectomy was 50% (*n* = 28/56), and an incidence of 30–60% in non-kidney solid organ tumor treatment was reported [5]. In adults, an incidence rate of 5% [29] to 47% [30] of AKI after kidney surgery has been reported, which is lower than in our pediatric cohort. This may be due to differences in surgical strategies, as most adult patients had renal cell carcinoma and underwent a partial nephrectomy without administration of chemotherapy [29, 30, 31, 32, 33, 34], while in our study, all but one patient underwent total nephrectomy. The higher AKI incidence in our pediatric cohort may partially result from the criteria used to define AKI, as pediatric (cancer) patients can meet AKI criteria with relatively small increases in creatinine levels [25].

We noticed that patients with AKI had a higher average baseline eGFR compared to patients who did not develop AKI. In line with this, studies in adults also indicated that patients at risk for AKI after radical nephrectomy had a higher eGFR in the preoperative phase [30, 31]. A higher preoperative GFR may indicate hyperfiltration. In case of hyperfiltration, a sudden decrease in kidney parenchymal mass may lead to a reduced extent of adaptation of GFR, making AKI more likely to occur in these patients [31].

This is supported by our intriguing observation that patients on average exhibited an elevation in eGFR during the initial weeks of preoperative chemotherapy. This novel finding has not been previously described in pediatric patients. It is unlikely that the hydration status of patients in this preoperative phase explains this, as most patients only visit the outpatient clinic for weekly pulses of chemotherapy without receiving hyperhydration. Prior reports stated that compensatory kidney enlargement might occur even before nephrectomy in individuals with contralateral renal carcinoma [35]. An increased eGFR and compensatory growth of the healthy kidney indicate hyperfiltration. To support this hypothesis in pediatric patients, evaluating contralateral kidney volume during the preoperative treatment period may be beneficial. Furthermore, CKD was observed in 13% (16/120) of the patients in our study. CKD was associated with the > 3-drug regimen, including ifosfamide and carboplatin, which are well-known risk factors for CKD in non-kidney tumor patients [36]. In contrast, CKD was not associated with radiation therapy, consistent with findings from other studies [37]. Additionally, CKD was not associated with genetic predisposing factors, contrary to other studies [38], likely due to the relatively short follow-up time. Interestingly, this is the first study that showed that AKI during pediatric kidney tumor treatment is associated with CKD, which is consistent with the established observation of a strong association between AKI and CKD in general [14] and more specifically after tumor nephrectomy among adults [30]. Our finding suggests that AKI after nephrectomy is not simply the predictable outcome of the loss of nephron mass, but rather through cell damage in the contralateral kidney. This hypothesis is supported by recent research showing significant changes in biomarkers of tubular injury, urine neutrophil gelatinase-associated lipocalin (NGAL), after radical nephrectomy [34], and evidence linking AKI after nephrectomy in adults to increased risk of CKD, morbidity, mortality, and prolonged hospitalization [29, 31, 39]_._ Therefore, early identification of AKI is important as this might be a modifiable risk factor. It warrants nephroprotective measures, such as discontinuation of nephrotoxic agents and timely dosing adjustments, optimization of volume status, and perfusion pressure to prevent further (permanent) deterioration of kidney function. In addition, intensive monitoring of functional hemodynamics, serum creatinine and urine output, avoidance of hyperglycemia, and consideration of alternatives for imaging procedures using radiocontrast is warranted [25]. Despite these nephroprotective measures, it stands to reason however that a sudden reduction in nephron mass contributes to the occurrence of AKI. Therefore, it holds significant value to ascertain the modifiability of this condition. Several authors have made efforts to adopt parenchymal-sparing surgical approaches, such as partial nephrectomy, where feasible and safe from an oncological perspective. Unfortunately, studies have reported a limited eligibility rate of only 4 to 8% among patients diagnosed with unilateral Wilms tumor for partial nephrectomy [40,41, 42]. To improve this, the current innovative development of image-guided surgery may lead to an increase in numbers of pediatric patients that benefit from oncological nephron-sparing surgery in unilateral tumors as well [43]. Ultimately, interventions should focus on preventing CKD, which is in turn an important risk factor for the occurrence of cardiovascular disease, impaired quality of life, and development and progression of disability [14].

Despite the large number of patients, this study is not without limitations with some missing data appropriate to the design of the study. First, as in almost all studies, sCr was adopted as an indicator of AKI despite its limitations due to the effects of nutritional and fluid status (dilutional effect of fluid overload) on sCR levels, and the non-daily ordering of sCr. The elevation of sCr is a slow and relatively insensitive indicator of AKI due to its manifestation up to 48–72 h following damage, and sCr can remain unchanged until more than half of the nephrons have lost their function [34]. This suggests that the incidence of kidney damage may be underestimated. This is supported by higher AKI incidence rates found in adults when using other biomarkers such as NGAL and cystatin C [34]. These biomarkers potentially enhance accuracy in detecting the severity and etiology of AKI. Secondly, establishing causality between AKI and CKD poses a challenge, as it remains uncertain whether AKI precipitates enduring kidney damage or AKI manifests because of pre-existing (genetic) kidney conditions. Although contralateral kidney volume was not reported, there were no differences in pre-existing eGFR between AKI and non-AKI groups. To further explain the post-nephrectomy AKI, it is imperative to implement a systematic monitoring approach encompassing kidney volumetrics and the application of biomarkers (such as NGAL) specifically linked to kidney injury in future pediatric oncology settings. In addition, long-term follow-up is needed to discover whether the association between AKI and CKD persists.

## Conclusion

AKI is a frequently (71%) observed complication of kidney tumor treatment in pediatric patients, occurring primarily following nephrectomy. Moreover, more than 13% of patients developed CKD within 1 year after completing therapy, with a higher prevalence among those who experienced AKI. Consequently, it is imperative to carefully monitor AKI during kidney tumor therapy and especially monitor these patients for proteinuria, hypertension, and decreased kidney function in long-term follow-up.

## Supplementary Information

Below is the link to the electronic supplementary material.

Graphical abstract (PPTX 140 KB)

Supplementary file1 (DOCX 613 KB)

## Data Availability

The data that support the findings of this study are available on request from the corresponding author. The data are not publicly available due to privacy or ethical restrictions.
