# Supplementary material for: The incidence and outcome of acute kidney injury during pediatric kidney tumor treatment—a national cohort study
Source: Pediatr Nephrol. 2025 Feb 19;40(7):2393–401. doi: 10.1007/s00467-025-06684-7 (PMC12116620; doi:10.1007/s00467-025-06684-7)
Supplement: Supplementary file 1 — Graphical abstract (PPTX 140 KB) [file 467_2025_6684_MOESM1_ESM.pptx]

## Slide 1
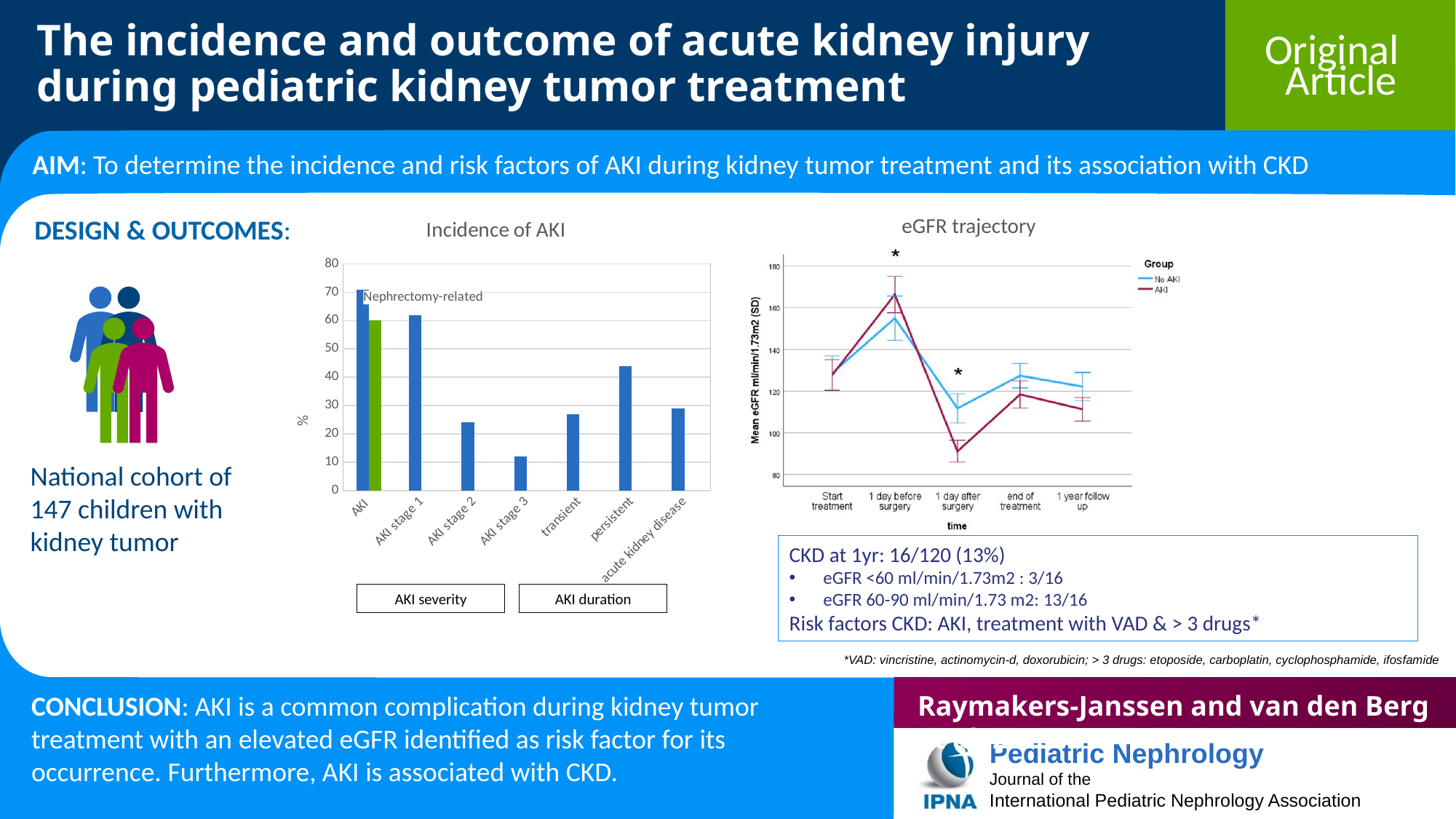

The incidence and outcome of acute kidney injury during pediatric kidney tumor treatment
AIM: To determine the incidence and risk factors of AKI during kidney tumor treatment and its association with CKD
### Chart: Incidence of AKI
| Category | Incidence of AKI, n=147 | nephrectomy-related |
|---|---|---|
| AKI | 71.0 | 60.0 |
| AKI stage 1 | 62.0 | None |
| AKI stage 2 | 24.0 | None |
| AKI stage 3 | 12.0 | None |
| transient | 27.0 | None |
| persistent | 44.0 | None |
| acute kidney disease | 29.0 | None |eGFR trajectory
DESIGN & OUTCOMES:
National cohort of
147 children with kidney tumor
CKD at 1yr: 16/120 (13%)
eGFR <60 ml/min/1.73m2 : 3/16
eGFR 60-90 ml/min/1.73 m2: 13/16
Risk factors CKD: AKI, treatment with VAD & > 3 drugs*
AKI severity
AKI duration
*VAD: vincristine, actinomycin-d, doxorubicin; > 3 drugs: etoposide, carboplatin, cyclophosphamide, ifosfamide
CONCLUSION: AKI is a common complication during kidney tumor treatment with an elevated eGFR identified as risk factor for its occurrence. Furthermore, AKI is associated with CKD.
Raymakers-Janssen and van den Berg et al. 2025
