# Supplementary material for: The incidence and outcome of acute kidney injury during pediatric kidney tumor treatment—a national cohort study
Source: Pediatr Nephrol. 2025 Feb 19;40(7):2393–401. doi: 10.1007/s00467-025-06684-7 (PMC12116620; doi:10.1007/s00467-025-06684-7)
Supplement: Supplementary file 2 — Supplementary file1 (DOCX 613 KB) [file 467_2025_6684_MOESM2_ESM.docx]

**Supplementary Material**

**The incidence and outcome of acute kidney Injury in children with a kidney tumor, a national cohort study**

*Paulien A.M.A. Raymakers-Janssen^1,2^ & *Gerrit van den Berg^2,3^, Inge A. van Kessel^1^, Marc Wijnen^2^, Mieke I. Triest^2^, Harm van Tinteren^2^, Geert O. Janssens^2,4^, Marc R. Lilien^3^, Marta Fiocco^2,5,6^, Roelie M. Wösten-van Asperen^1^, Marry M. van den Heuvel-Eibrink^2,7^

**Supplementary Table 1** Demographics of kidney tumor patients

**Supplementary Table 2**  Characteristics of patients with non-direct kidney surgery-related AKI

**Supplementary Figure 1** Incidence of acute kidney injury per Wilms tumor stage, at different timepoints during treatment

**Supplementary Figure 2**  Details of AKI severity and AKI duration

**Supplementary Table 1. Demographics of kidney tumors**

| **Disease** | **N** | **Median age in months (IQR)** | **Stage** | | | | | | | | **Surgery** | | |
| --- | --- | --- | --- | --- | --- | --- | --- | --- | --- | --- | --- | --- | --- |
|  |  |  | localized | | |  | metastatic | | | NA | N | NSS | Bilateral |
|  |  |  | I | II | III | V | I | II | III |  |  |  |  |
| WT | 126 | 36 (21-54) | 34 | 27 | 23 | 17 | 4 | 4 | 17 |  | 117 |  | 9 |
| CN | 5 | 15 (11-51) | 2 |  |  |  |  |  |  | 3 | 3 | 1 | 1 |
| CMN | 5 | 2 (0 – 4) |  | 4 | 1 |  |  |  |  |  | 5 |  |  |
| RCC | 6 | 145 (64-187) | 3 | 1 |  |  |  |  |  | 2 | 6 |  |  |
| MRTK | 2 | 3;6 |  | 2 |  |  |  |  |  |  | 2 |  |  |
| MA | 1 | 21 |  |  |  |  |  |  |  | 1 | 1 |  |  |
| Nephrogenic rest | 2 | 13;20 |  |  |  |  |  |  |  | 2 | 2 |  |  |
| **Total** | 147 | 35 (0-227) | 39 | 34 | 24 | 17 | 4 | 4 | 17 | 8 | 136 | 1 | 10 |
| WT: Wilms tumor, CN: cystic nephroma, CMN: congenital mesoblastic nephroma, RCC: renal cell carcinoma, MRTK: malignant rhabdoid tumors of the kidney. MA: metanephric adenoma, NA: not applicable, N: nephrectomy, NSS: nephron sparing surgery, Bilateral = N + NSS or NSS both sides. | | | | | | | | | | | | | |

**Supplementary Table 2. Characteristics of patients with non-direct kidney surgery-related AKI**

| **Clinical characteristics (sex, age, stage, histology)** | **Clinical condition suspected to be associated with AKI risk as assessed by pediatric oncologist** | **Underlying problem/ genetic predisposing factor.*** |
| --- | --- | --- |
| **AKI from diagnosis before nephrectomy** | | |
| Male 3.9y, WT stage IV IR | Tumorthrombus vena cava inferior and right atrium. Hypertension. | x |
| Female, 1.6 y, WT stage II IR | Dehydration at diagnosis. Antihypertensive drugs. | x |
| Male, 2.6y, WT stage I, IR | Postrenal obstruction, macroscopic hematuria | x |
| Female, 4.1y, WT stage IV, LR | Volume depletion due to vomiting and insufficient intake | x |
| Male,, 2.6y WT stage IV IR | Volume depletion due to vomiting, insufficient intake. Tumor thrombus vena cava inferior. | x |
| **AKI more than 48 hours after nephrectomy** | | |
| Female, 5.3y, RCC, stage I | No reason | x |
| Female, 6.1y, WT stage I, LR | Volume depletion due to gastro-enteritis | x |
| Female, 1.3y, WT stage V (I), HR-BP | Volume depletion due to gastro-enteritis. Pre-existent hypertension | REST-mutation |
| Male, 3.8y, WT stage IV IR | Resection of tumor thrombus from right atrium | x |
| Female, 2y, WT stage I IR | Volume depletion due to feeding problems | Beckman Wiedeman spectrum |
| Male, 3.8, WT stage V IR | Urinary leakage after nephron sparing surgery | Nephron sparing surgery left side after total nephrectomy right |
| Female, 1.4y, WT stage II IR | Volume depletion and use of ACE-inhibition | CKD stage 1 due to Fanconi anemia FANCD1/BRCA2-mutation |
| Female, 1.7y, nephrogenic rest | Volume depletion due to gastro-enteritis | Hemihypertrophy |
| Male, 4,.1y, WT stage IV +V HR | Three AKI episode after ICE-therapy | Beckman Wiedeman spectrum |
| Female, 4.3 y, WT stage III IR | Volume depletion due to insufficient intake and ICE-therapy | x |
| Female, 3.1y, WT stage III IR | nausea and vomiting after VA,  use of ambisome after ICE-therapy. | *DIS3L2-*mutation |
| AKI: acute kidney injury; CKD: chronic kidney disease,; LR: low risk; IR: intermediate risk; HR-BP: high risk blastemal predominant; V: vincristin; A:actinomycin-D; D: doxorubicin; ICE: ifosfamide, carboplatin, etoposide. *every patient (if parents consented) was tested for Beckman Wiedeman spectrum and underwent WES based panel (in case of Wilms tumor) | | |

**Supplementary
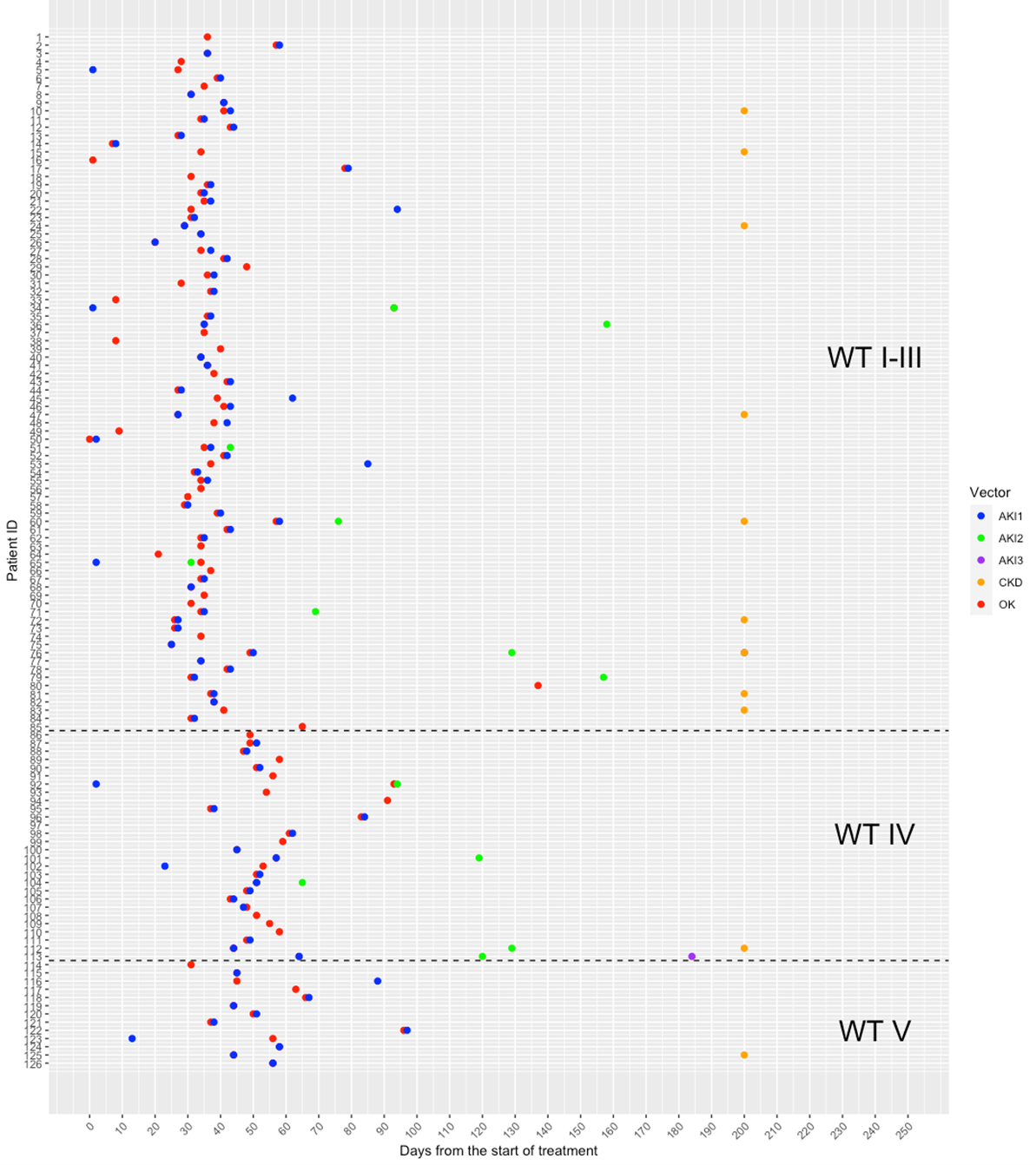
Figure 1. Incidence of acute kidney injury per Wilms tumor stage, at different timepoints during treatment**

*The incidence of AKI and CKD in the126 Wilms tumor patients during treatment. WT: wilms tumor; I-III: stage 1,2,3; IV; stage 4; V: stage 5. AKI: acute kidney injury; OK; surgery (nephrectomy); CKD: chronic kidney disease.*

**Supplementary Figure 2. Details of AKI severity and AKI duration**

AKI severity

AKI duration

*I*
